# Future-oriented thinking promotes positive attitudes toward the “Help Mark” in Japan

**DOI:** 10.3389/fresc.2022.967033

**Published:** 2022-11-17

**Authors:** Hirofumi Hashimoto, Kaede Maeda, Kosuke Sato

**Affiliations:** ^1^ Graduate School of Literature and Human Sciences, Osaka Metropolitan University, Osaka, Japan; ^2^ Department of Psychology, College of Contemporary Psychology, Rikkyo University, Niiza, Japan; ^3^ Department of Psychology, Faculty of Literature, Kurume University, Kurume, Japan; ^4^ Center for General Student Support, Kochi University, Kochi, Japan

**Keywords:** disabilities, rehabilitation science, hidden disability, Help Mark, future-oriented thinking, attitude, prejudice

## Abstract

The “Help Mark,” created in Japan, is worn by people who need help in public settings. It is designed to induce help from others for those in need of help because of their hidden disabilities or health conditions. Several attempts have been made to publicize the meaning and implications of this wearable sign through various media. However, it is difficult to assert whether there is sufficient awareness regarding this sign in the Japanese society. The purpose of this study was to examine the type of messages that are more effective in promoting the “Help Mark” system (Study 1). Additionally, based on the data obtained in Study 1, we presented a newly designed poster to promote the “Help Mark” sign and attempted to empirically examine the effect of this poster (Study 2). The results suggest that a message that reflects that the “Help Mark” is for “everyone,” based on future-oriented thinking, is more effective. Furthermore, it was indicated that people who saw the poster containing a message implying that the “Help Mark” is “for everyone” reported increased positive attitudes toward the “Help Mark” system. These results indicate that encouraging future-oriented thinking may lead to positive attitudes regarding the “Help Mark” system.

## Introduction

According to the World Health Organization (WHO), approximately 15% of the global population or over one billion individuals live with one or more disabling conditions (1); notably, most of these conditions are *invisible*. Invisible disability is a physical, mental, or neurological condition that is hidden or not visible to an observer. There is not enough data about the rate of prevalence of invisible disabilities. However, it is easy to imagine that the rate is higher than expected as studies show that about 10% of Americans have a medical condition, which could be considered an invisible disability and 96% of those with chronic medical conditions live with a condition that is invisible (2). Besides chronic medical conditions, there are many invisible disabilities such as neurodevelopmental disorders, mental disorders, hearing impairments, and symptoms such as chronic pain, fatigue, and dizziness.

To promote inclusion of people with hidden and invisible disabilities and ensure their rights and participation in the society is an urgent issue for countries that ratified the Convention on the Rights of Persons with Disabilities (CRPD) (3), including Japan. In this study, we focus on a unique system to promote social inclusion for people with hidden or invisible disabilities in Japan called the “Help Mark.” Despite its proposed usefulness, the recognition and effectiveness of the “Help Mark” among the general public is low (4). Our study examines whether future-oriented thinking increases positive attitudes toward this system.

### The “Help Mark” and its current status

**Figure 1:**
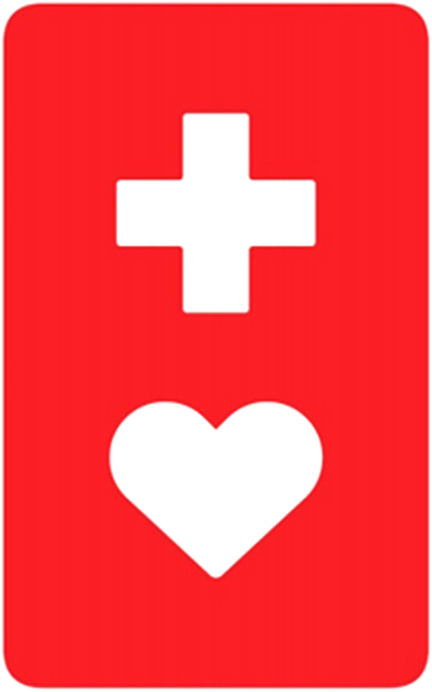
The Help Mark.

In Japan, a unique system has been developed to increase inclusion of people with disabilities in public places. People with disabilities or chronic health conditions, particularly invisible disabilities, who might need help can wear a badge called the “Help Mark” (see Figure 1). This sign depicts a white cross and a heart on a red background. The red background and the cross are meant to indicate “help needed,” and the heart symbol to indicate “willingness to help.”

The “Help Mark” was originally created in 2012 by Akemi Yamaka, a member of the Tokyo Metropolitan Assembly who has artificial joints, to make the daily lives of people with “hidden disabilities” (e.g., people with prosthetic legs, artificial joints, internal ailments, and internal and rare diseases) easier by receiving assistance from people around them in public settings. This system was designed to induce help from others and to create an inclusive environment for people with invisible disabilities. The Tokyo Metropolitan Government distributes the “Help Mark” badges at metropolitan transportation facilities (such as subway stations and bus offices). Posters have also been displayed in public transport and other places to promote awareness of this sign.

The “Help Mark” is being promoted as a part of efforts to develop a symbiotic society. The Ministry of Economy, Trade and Industry (METI) made further efforts to promote awareness of this wearable sign by revising the JIS Z8210 standard for graphical symbols for guidance, with the aim of making this symbol easier to understand not only for the Japanese but also for foreign tourists, in preparation for the 2020 Tokyo Olympics and Paralympics. As a result, public institutions as well as local and prefectural governments attempted to promote this sign in Japan. The “Help Mark” thus became a nationwide sign and system (5).

To date, however, it has been difficult to assert that these attempts by public institutions have sufficiently penetrated the Japanese society. In spite of the importance and implications of this system, the sign is still not recognized by many. According to a survey by the *Shougaisha* (Japanese for “people with disabilities”) Research Institute on the prevalence of the “Help Mark,” approximately 47% of the respondents had knowledge of this sign. Furthermore, 55% of respondents in the Greater Tokyo Area knew about this mark, while only 38% in other regions knew about it, indicating insufficient awareness about it in regions other than the Greater Tokyo Area (4). These data also indicated that people hesitate to use this sign, and one of the main reasons for this is that they fear that other people's reactions would not be what they expect because of the lack of recognition and understanding of the sign. Therefore, this study aims to promote awareness of this sign among the general public.

### Future-oriented thinking

Before a specific examination through a survey or field observation, we began with an examination of the current problem of low public recognition of the sign and considered the wording of the message in almost all posters related to the “Help Mark” as points for improvement. The current message is that the “Help Mark” is for those who need assistance. Such a message implicitly makes a clear distinction between the position of those who help and those who need help. Therefore, some people may be hesitant to help and feel that it is more costly than necessary (6, 7). However, it is important to consider that social welfare is not about the tradeoff of costs and benefits in the “here and now.” On the contrary, social welfare is a part of preparation for the day that will come for everyone. In the long run, social welfare functions as an important “insurance” for oneself and one's family in the future.

Based on the arguments of Baumeister et al. (8), people's beliefs about the future are beneficial in that current decisions can be guided by numerous possibilities (a matrix of “maybes”). In other words, decisions made in the “here and now” that are based on thought processes trapped in the present can also be determined in a way that considers benefits based on a longer-term perspective. Such benefits of future-oriented thinking were confirmed by Vonasch and Sjåstad (9), who suggested that manipulating future orientation may lead people to focus on benefits that are based on a longer-term perspective, rather than on short-term benefits. Maeda et al. (10) also demonstrated that thinking about the future employment of people with/without disabilities has encouraged positive attitudes toward inclusive education, which is a major challenge for educational systems worldwide. Such future-oriented thinking is useful for social welfare, because the evaluation of the tradeoff between the benefits and costs of social welfare can depend on how people perceive the time axis; the frame of “for whom does the Help Mark exist” differs depending on whether one is taking a short- or long-term perspective. More specifically, if the costs and benefits are understood in the short-term, and the “Help Mark” is understood as being only for people who need help (i.e., present-oriented thinking), then the importance and implications of the mark will be difficult to demonstrate or explain. Although if the costs and benefits are considered in the long-term perspective, and the “Help Mark” is perceived as being for “everyone” by creating an environment that makes it easier to obtain support in the long run (i.e., future-oriented thinking), public recognition of its importance will increase.

### The current study

Based on the arguments listed above, two studies were conducted to examine the prevalence and understanding of the “Help Mark.” In Study 1, we conducted a survey based on the hypothesis that conveying the message that the “Help Mark” system is for “everyone” will result in positive attitudes toward the sign. In Study 2, based on the results of Study 1, we conducted a field observation to confirm whether the above-mentioned message actually improved positive attitudes toward the sign. The purpose of Study 2 was to confirm the robustness and applicability of our findings.

## Materials and methods

### Study 1

#### Methods

After approval from the Ethics Committee, Study 1 was conducted as part of a lecture on introduction to social research. One hundred and twenty-one female Japanese university students (mean age = 20.2 years) participated in this study. Participants were assigned to three different conditions. They were all asked to fill out a consent form and choose one of the envelopes (placed in a box in a jumble) prepared for each of the three conditions. In these envelopes, participants were randomly given a questionnaire containing a description of the “Help Mark”; three types of descriptions were prepared for the three conditions. For participants assigned to the control condition (*n* = 40), only the definition of the “Help Mark” was explained. For participants assigned to the present-oriented thinking condition (*n* = 40), in addition to the definition, it was emphasized that the “Help Mark” is a sign for those who need support and that those who provide support will attain peace of mind. These descriptions were referenced from posters that are displayed in many public institutions in Japan. In the future-oriented thinking condition (*n* = 41), in addition to the definition, it was emphasized that the “Help Mark” is a sign for everyone, not just those who need help, and that creating a society in which people can easily help each other will lead to safety for all people including oneself in the long run. The participants were asked to read these descriptions carefully for 3 min, and then asked to answer two items on a seven-point scale for a manipulation check: (1) “Do you think that the “Help Mark” is a sign for ‘people who need support’?” and (2) “Do you think that the “Help mark” is a sign for ‘people who provide support’?” The score for the second question was likely to be higher under the future-oriented thinking condition. Subsequently, participants were also asked to answer questionnaire items about their positive perceptions of the “Help Mark” (seven items; e.g., “I think the ‘Help Mark’ needs to be more widely used in society,” Cronbach's *α* = .81) and items to measure their reluctance (three items; e.g., “It is unacceptable that only those who use the ‘Help Mark’ are given preferential treatment,” *α* = .70), using a seven-point scale. The specific scale items are shown in Supplementary Table S1.

#### Results

Mean scores of the items used for manipulation check by condition are shown in Supplementary Table S2. First, we performed Kruskal–Wallis tests, with the conditions as independent variables and the scores of the items used for manipulation check as the dependent variables, and found the statistically significant differences for both scores (whether they think it is for “people who need support”: *χ*^2^(2) = 8.54, *p* = 0.01, *η*^2^ = .07; whether they think it is for “people who provide support”: *χ*^2^(2) = 13.71, *p* < 0.01, *η*^2^ = .11). A *post hoc* pairwise comparison showed that there was a significant difference between the control condition and the future-oriented condition (*Dwass–Steel–Critchlow–Fligner pairwise comparison*, *p* < 0.01) in the item about whether they think it is for “people who need support.” Despite differences between the conditions, a high level of positive perception was confirmed. We also found a significant difference between the control condition and the future-oriented condition (*p* < 0.01), and a significant difference between the present-oriented thinking condition and the future-oriented condition (*p* < 0.01) in the questionnaire item about whether they think it is for “people who provide support.” The results showed that by manipulating the participants’ future orientation, we could successfully enhance their perception that the “Help Mark” exists for “everyone,” not for only people need help from others.

**Figure 2:**
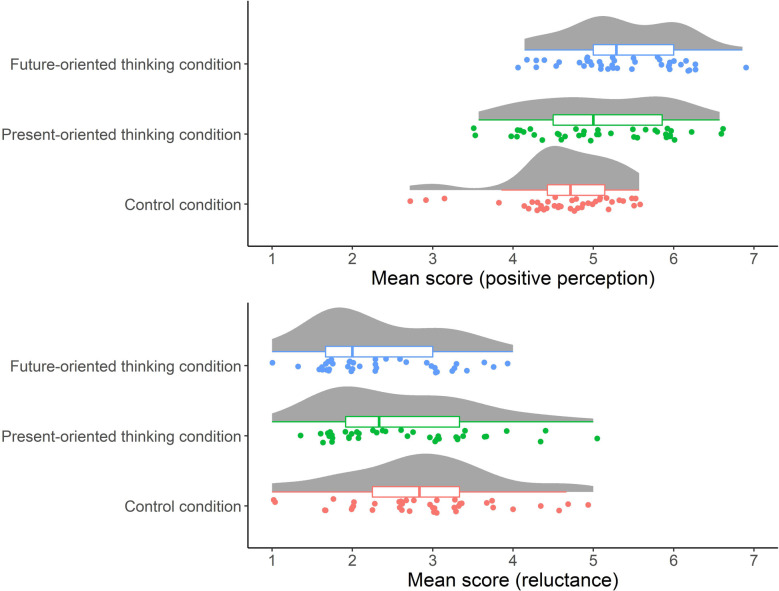
Distributions of the two scale scores by conditions in Study 1. Each point represents each participant. Boxplots indicate the distributions of the two scale scores. Random vertical jitter was added to each for ease of visibility.

We then conducted an exploratory factor analysis with Promax rotation using questionnaire item scores about their positive perceptions of the “Help Mark” and potential reluctance. As predicted, the analysis yielded two factors. Therefore, the mean scores for each scale were calculated and used in the analysis. The concrete scale items and factor loadings are shown in Supplementary Table S1 and mean scores of the positive perception scale and reluctance scale are shown in Supplementary Table S2. Furthermore, the distributions of the two scale scores by conditions are shown in Figure 2. To examine the effect of condition, we performed ANOVA with the scores of the positive perception scale. The result demonstrated that there was a main effect of condition: positive perception [*F* (2, 118) = 9.96, *p* < 0.001, $\eta_{p}^{2}=0.14$]. To clarify the main effect of condition, we performed a multiple comparison analysis and found a significant difference in the positive perception scores between the control and present-oriented thinking conditions [*t* (118) = 2.75, *p* < 0.01, *d *= 0.61] and between the control and future-oriented thinking conditions [*t* (118) = 4.42, *p* < 0.001, *d* = 0.97]. We also performed Kruskal–Wallis tests with the scores of the reluctance scale and found significant difference [*χ*^2^(2) = 7.56, *p* < 0.05]. An additional multiple comparison analysis revealed a significant difference [*p* < 0.01] in the reluctance scores between the control and future-oriented thinking conditions (see Figure 2).

#### Discussion

In Study 1, we examined whether the message that the sign is for everyone could change people's positive perception or reluctance, or both, toward the “Help Mark.” As shown in Figure 2, compared with the control condition, participants in the future-oriented thinking condition had higher scores for positive perception and lower scores for reluctance. These results support our hypothesis and suggest that thinking about one's future as a concerned person leads to a more tolerant understanding of the “Help Mark.” However, it is not clear to what extent these results can be applied to real life settings. Therefore, based on the findings of Study 1, in Study 2, we conducted a field observation study to test whether a new poster with a message that the “Help Mark is for everyone” increased participants’ recognition and understanding of this system.

### Study 2

Based on the results of Study 1, we designed a new poster to enlighten people about the fact that the “Help Mark” is for everyone, with the staff from Hiroshima Rapid Transit Co., Ltd., operating the automated people mover called “Astram Line” in Hiroshima, Japan (Study 2 was conducted as part of a collaborative project with the Hiroshima Rapid Transit Co., Ltd., and the posters we created were actually used for public awareness). After approval from the Ethics Committee, we conducted the field observation study where we displayed the poster in a women's university for about 1 month and examined whether peoples’ perceptions can be changed. In the poster, we displayed a woman walking with the “Help Mark” badge on her bag and added the following sentence: “Even if you don’t need help now, there may come a day in the near future when you or someone you really care about will need help from those around you.” We also added the following: “Attempting to create a supportive society will lead to an assurance system for everyone, including you, in the future.” These sentences were added to make university students who saw the poster realize that the “Help Mark” system would be beneficial for everyone in the long run.

#### Methods

In Study 2, we recruited 92 female Japanese undergraduates (mean age = 18.9 years) from a lecture in the Department of Psychology. This experiment was conducted over 2 weeks. Before presenting our newly designed poster in our university (week 1), the participants were asked to answer three items for manipulation checks. These included the same two questions from Study 1, using a seven-point scale: (1) “Do you think that the ‘Help Mark’ is a sign for ‘people who need support’?” and (2) “Do you think that the ‘Help Mark’ is a sign for ‘people who provide support’?” We added one more item: (3) “Do you think that the ‘Help Mark’ is a sign for ‘everyone,’ including you, in the future in the long run?” The scores for the second and third questions were expected to be higher if the participants had seen the poster that emphasized that the “Help Mark” is for everyone in a long run. Subsequently, participants were also asked to answer questionnaire items about their positive perceptions of the “Help Mark” (seven items; *α*_week1_ = .75, *α*_week2_ = .79) and questionnaire items to measure their reluctance (three items; *α*_week1_ = .80, *α*_week2_ = .67), using a seven-point scale, as in Study 1 (see Supplementary Table S1). After 1 month, similar to the task in week 2, participants were again asked to answer the same questionnaire items used in week 1. In the questionnaire items in week 2, we also asked the participants whether they had actually seen the poster displayed in the university or not. In Study 2, the data were analyzed primarily to determine a change in attitudes and perception between those who had seen the poster and those who had not. We calculated the difference between the scores (week 2—week 1) as the main dependent variable.

#### Results

**Figure 3:**
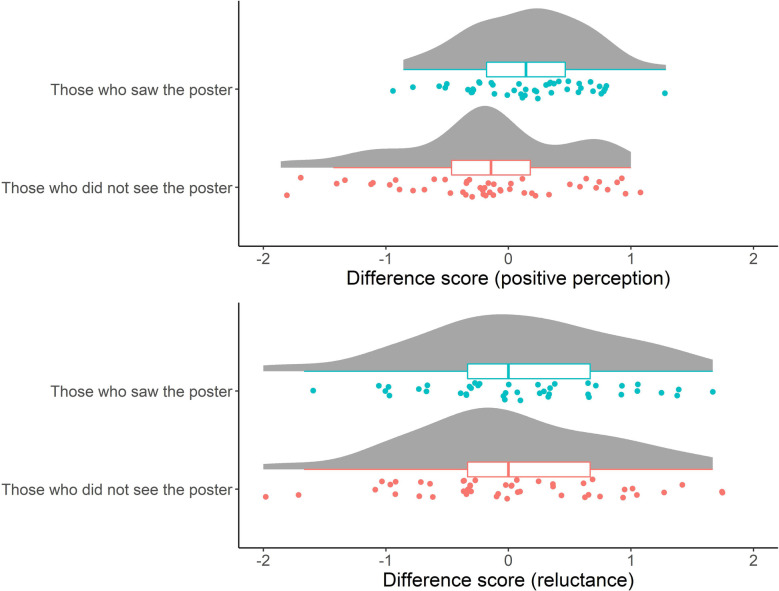
Distributions of the difference in the two scale scores between the two weeks (pre and post) among those who saw the poster and those who did not. Each point represents each participant. Boxplots indicate the distributions of the two scale scores. Random vertical jitter was added to each point for ease of visibility.

First, of the 92 participants, 44 confirmed that they had seen the poster, while 48 said that they had not. The descriptive statistics regarding the items used for manipulation check and the two (positive perception and reluctance) scales, among those who saw the poster and those who did not, are shown in Supplementary Table S3. As shown in Supplementary Table S3, it was found that among those who saw the poster, the perception that the mark is for “everyone” increased more in the second week compared with the first week (*Wilcoxon signed rank test*, *p* = 0.01). Furthermore, Figure 3 shows the distributions of the difference in the two scale scores between the two weeks (pre and post) among those who saw the poster and those who did not. The difference in difference scores among those who saw the poster and those who did not was significant [*t* (90) = 2.85, *p* < 0.01]; the positive perceptions were found to be significantly higher only among those who had seen the poster (*M* = 0.16). A one-sample t-test of this difference score was conducted as an additional exploratory analysis and showed that this score was significantly above the theoretical median (0) [*t* (43) = 2.22, *p* = 0.03]. However, no significant difference was found for reluctance.

#### Discussion

In Study 2, we examined whether a newly designed “Help Mark” poster increased positive perception of the “Help Mark” and its system through a field observation study. The results showed that positive perceptions toward this sign increased among students who saw the poster, and the perception that this system is for everyone also increased. These results indicate the effectiveness of messages that encourage people to think about their future as a concerned person, by envisaging the possibility that they or someone in their family may need to use the “Help Mark” in the future.

## General discussion and conclusion

In order to determine more effective ways of publicizing the “Help Mark” and encouraging its use, Study 1 was conducted with the hypothesis that conveying a message that the “Help Mark” is a sign for everyone would increase positive perception toward the mark. The hypothesis was supported, and it was suggested that such a message may be an effective way to promote awareness and understanding of the sign. Furthermore, in Study 2, a new poster was created to promote the “Help Mark” and a field observation study was conducted to measure its effectiveness. The results indicated the effectiveness of conveying the message that the “Help Mark” is “for everyone” based on future-oriented thinking. Specifically, it was found that people who saw the new poster showed increased positive perception toward the “Help Mark” system.

Recently, significant efforts have been undertaken in Japan regarding the prevalence and effectiveness of the “Help Mark” and the promotion of peoples’ understanding of social inclusion of those who are socially vulnerable. The results of this study suggest that thinking about the future as a concerned person may hold the key to publicizing the “Help Mark” effectively, which indicates the usefulness and applicability of conveying such messages; in this sense, our results have a strong potential for contribution in this field. However, it is necessary here to consider the discrimination against disability (both visible and invisible) and the labeling and stigmatization of people with disabilities. Undeniably, the Help Mark may provoke prejudice and labeling against people with disabilities if a proper understanding of disability is not widespread. Suppose the possibility of such prejudice and labeling makes people with disabilities hesitant to utilize the “Help Mark,” and, as a result, the “Help Mark” is not well known to the public. In that case, this will go against the original purpose of the “Help Mark.” What is needed now is to promote a correct understanding of disability and make people aware of the potential value of the “Help Mark.” Especially for the Japanese society, which is considered to have difficulties in realizing an inclusive society (11), it is important to simultaneously increase the correct understanding of people with disabilities and awareness of the “Help Mark,” and this study proposes one concrete method to do so.

However, this study has some limitations. First, it is potentially problematic that our results are based on a sample limited to young Japanese female students. Additionally, the positive perception and reluctance scales that we administrated in this study should be considered a preliminary scale. Future study should confirm the robustness of our findings. Second, the results of Study 2 did not identify causality. Notwithstanding the importance of field observations, a more rigorous examination of the potential effects of the message is required. Third, it is possible that the study manipulated both future-oriented thinking itself and perceptions of future benefits for participants. Future studies, therefore, should manipulate future orientation only and examine which factor—future-oriented thinking or perceptions of future benefits as a concerned person—is more effective. Finally, although the present study focuses only on the psychology of those who notice and support a person wearing the “Help Mark,” in future, it is necessary to analyze the psychology of those who wear the sign themselves in the same way. In doing so, it is necessary to carefully analyze their perceptions of stigmatization, discrimination, and labeling, for both visible and invisible disabilities.

Reflecting on an effective way to improve the use of the “Help Mark,” one should consider the fact that publicizing this sign in such a way is a phenomenon unique to the Japanese society. Although it is possible in an ideal world to have a society where people support each other without such signs, it is important to consider that Japanese people may not engage in such prosocial behaviors often. This tendency may be more robust toward people with disabilities. According to a cross-cultural study about social inclusion for people with disabilities in Japan, Germany, and the United States by the Japanese government (12), a majority of Japanese people were found to not treat people with disabilities as usual. Conversely, most of the people in Germany and the United States treated people with disabilities in the usual manner. Research in cultural psychology (13) has shown that social support is less likely to be sought and provided in East Asian societies (14–16). A possible reason for this is that the sense of “relational concern” (14, 17) is more pronounced among East Asians, who tend to be reluctant to seek social support to cope with their difficulties and stress because they are concerned that their accruing potentially negative reputation could burden their group harmony (17). Related studies have also argued that Japanese people tend to avoid being disliked or rejected by the people around them (18–20), which makes it difficult for them to voluntarily help or seek help from each other. They should, therefore, be encouraged to support each other, using the “Help Mark” system. In other words, the “Help Mark” has the potential to encourage mutual help in the Japanese society. Within this context, it is extremely important to understand how the “Help Mark” is conveyed to and understood by Japanese people. If this mark is portrayed as a sign that clearly distinguishes those who need help and those who help, the social implication of the “Help Mark” may be insignificant. However, if, as the current study suggests, the mark is portrayed as a sign that will benefit everyone in the long run, its social implication may be substantial. The present study, which shows the effectiveness of the latter from the perspective of applied social psychology, can thus be considered significant in this sense.

## Data Availability

The datasets presented in this study can be found in online repositories. The names of the repository/repositories and accession number(s) can be found here: https://osf.io/5xkts/?view_only=50563ec64540436680142f65b96b6eb6.
