# Supplementary material for: Future-oriented thinking promotes positive attitudes toward the “Help Mark” in Japan
Source: Front Rehabil Sci. 2022 Nov 17;3:967033. doi: 10.3389/fresc.2022.967033 (PMC9712963; doi:10.3389/fresc.2022.967033)
Supplement: Supplementary file 1 [file TableS1.pdf]

Table S1. Descriptive statistics and factor loadings (EFA and CFA) of the positive perception scale and reluctance scale

	Study 1				Study 2 (week 1)				Study 2 (week 2)			
	EFA		Means and standard deviations		CFA		Means and standard deviations		CFA		Means and standard deviations	
	F1	F2	M	SD	F1	F2	M	SD	F1	F2	M	SD
**Factor 1: Positive perceptions**												
I would let the people around me know about the Help Mark.	**.767**	.243	3.95	1.34	**.391**		3.54	1.33	**.619**		3.60	1.42
I would like to know more about the Help Mark.	**.744**	.182	4.00	1.28	**.679**		5.12	1.08	**.621**		5.13	1.13
I would be more concerned about people who use the Help Mark.	**.615**	-.202	5.46	0.92	**.583**		4.91	1.40	**.837**		4.87	1.29
I would talk to people using the Help Mark if they were in need.	**.590**	-.041	4.81	1.31	**.593**		4.98	1.31	**.391**		4.95	1.37
I think that the help mark needs to be more widely spread in society.	**.563**	-.177	6.04	0.86	**.786**		5.79	1.00	**.678**		5.67	1.05
In the case of a disaster, I would help people with the Help Mark to evacuate safely.	**.551**	-.105	5.29	1.04	**.516**		4.76	1.42	**.597**		4.65	1.46
I would give up my seat on trains and buses to people with Help Mark.	**.470**	-.164	5.65	1.05	**.311**		3.30	1.36	**.381**		3.35	1.39
**Factor 2: Reluctance**												
I am not satisfied that only people with the Help Mark are given preferential treatment.	.012	**.759**	1.98	1.06		**.699**	2.01	1.14		**.761**	1.99	1.12
I think that if we improve care and assistance for people with help marks, the load on the people who provide care will increase.	.070	**.727**	3.02	1.21		**.736**	3.34	1.49		**.524**	3.34	1.42
To be honest, I would like to put as much distance as possible between myself and people who use the Help Mark.	-.128	**.495**	2.70	1.20		**.823**	2.45	1.23		**.681**	2.60	1.42
