# Supplementary material for: Future-oriented thinking promotes positive attitudes toward the “Help Mark” in Japan
Source: Front Rehabil Sci. 2022 Nov 17;3:967033. doi: 10.3389/fresc.2022.967033 (PMC9712963; doi:10.3389/fresc.2022.967033)
Supplement: Supplementary file 2 [file TableS2.pdf]

Table S2. Mean scores of the items used for manipulation check and the positive perception scale and reluctance scale by condition (Study 1).

	Perception that the mark is for "people who need support"	Perception that the mark is for "people who provide support"	Positive perceptions	Reluctance
Future-oriented thinking condition (n=41)	6.56 (0.63)	5.54 (1.43)	5.35 (0.67)	2.28 (0.78)
Present-oriented thinking condition (n=40)	6.48 (0.85)	4.10 (2.13)	5.09 (0.83)	2.59 (0.93)
Control condition (n=40)	6.13 (0.76)	4.38 (1.64)	4.64 (0.65)	2.84 (0.97)

Note. Standard deviations are indicated in parentheses.
