# Supplementary material for: Future-oriented thinking promotes positive attitudes toward the “Help Mark” in Japan
Source: Front Rehabil Sci. 2022 Nov 17;3:967033. doi: 10.3389/fresc.2022.967033 (PMC9712963; doi:10.3389/fresc.2022.967033)
Supplement: Supplementary file 3 [file TableS3.pdf]

Table S3. Mean scores of the items used for manipulation check and the positive perception scale and reluctance scale by condition (Study 2).

		Perception that the mark is for "people who need support"	Perception that the mark is for "people who provide support"	Perception that the mark is for "everyone"	Positive perceptions	Reluctance
Those who saw the poster (n=44)	Week 1	6.05 (0.99)	4.09 (1.93)	4.61 (1.50)	4.40 (0.87)	2.69 (0.99)
	Week 2	5.82 (1.17)	4.57 (1.73)	5.18 (1.51)	4.55 (0.87)	2.73 (0.74)
	Difference score	-0.23 (1.43)	0.48 (2.17)	0.57 (1.39)	0.16 (0.47)	0.05 (0.97)
Those who did not see the poster (n=48)	Week 1	6.00 (1.24)	4.17 (1.96)	5.48 (1.37)	4.85 (0.68)	2.51 (1.19)
	Week 2	5.52 (1.74)	4.10 (1.81)	5.15 (1.29)	4.65 (0.87)	2.56 (1.24)
	Difference score	-0.48 (1.58)	-0.06 (1.79)	-0.33 (1.67)	-0.20 (0.69)	0.04 (1.01)

Note. Standard deviations are indicated in parentheses.
